# Effects of Cell Proteostasis Network on the Survival of SARS-CoV-2

**DOI:** 10.1186/s12575-021-00145-9

**Published:** 2021-02-22

**Authors:** Fateme Khomari, Mohsen Nabi-Afjadi, Sahar Yarahmadi, Hanie Eskandari, Elham Bahreini

**Affiliations:** 1 Department of Biochemistry, Faculty of Medicine, Iran University of Medical Sciences, P.O. Box: 1449614525, Tehran, Iran; 2 Department of Biochemistry, Faculty of Biological Science, Tarbiat Modares University, Tehran, Iran; 3 Department of Biology, Science and Research Branch, Islamic Azad University of Tehran, Tehran, Iran

**Keywords:** Coronavirus, COVID-19, SARS-CoV-2, Chaperone, Proteostasis

## Abstract

The proteostasis network includes all the factors that control the function of proteins in their native state and minimize their non-functional or harmful reactions. The molecular chaperones, the important mediator in the proteostasis network can be considered as any protein that contributes to proper folding and assembly of other macromolecules, through maturating of unfolded or partially folded macromolecules, refolding of stress-denatured proteins, and modifying oligomeric assembly, otherwise it leads to their proteolytic degradation. Viruses that use the hosts’ gene expression tools and protein synthesis apparatus to survive and replicate, are obviously protected by such a host chaperone system. This means that many viruses use members of the hosts’ chaperoning system to infect the target cells, replicate, and spread. During viral infection, increase in endoplasmic reticulum (ER) stress due to high expression of viral proteins enhances the level of heat shock proteins (HSPs) and induces cell apoptosis or necrosis. Indeed, evidence suggests that ER stress and the induction of unfolded protein response (UPR) may be a major aspect of the corona-host virus interaction. In addition, several clinical reports have confirmed the autoimmune phenomena in COVID-19-patients, and a strong association between this autoimmunity and severe SARS-CoV-2 infection. Part of such autoimmunity is due to shared epitopes among the virus and host. This article reviews the proteostasis network and its relationship to the immune system in SARS-CoV-2 infection.

## Introduction

Since 2019, severe acute respiratory syndrome coronavirus-2 (SARS-CoV-2) infection, the seventh human coronavirus infectious disease, has affected about 218 countries and territories and caused the deaths of hundreds of thousands of people around the world. It causes coronavirus disease 2019 (COVID-19) with high rates of infection and severe respiratory disorders characterized by early symptoms of fever, cough, sputum production, fatigue, and myalgia. SARS-CoV-2 is an enveloped, positive-sense, single-stranded RNA virus with a genome of nearly 30,000 nucleotides containing 5′ capped and 3′ polyadenylated. Entry into host cells is the first step of viral infection that is occurred through interaction of viral spike glycoprotein and angiotensin-converting enzyme 2 (ACE2) receptor. High infection of the virus due to high rate of its proliferation, naturally leads to mutations in its genome, which, if the host protein synthesis, maturation and modification systems work together, leads to more aggressive forms of the virus and greater resistance [1]. Like other viruses, SARCE-CoV-2 mutates over time to adapt. Indeed, thousands of mutations have occurred since the virus was identified [2]. The genome of RNA viruses is highly susceptible to random mutations, due to the lack of proofreading activity in their viral RNA polymerase [3]. The resulting variance in the protein sequence may lead to changes in the structure and function of the protein that strengthen viral survival capability, pathogenicity and epidemics. The survival of the virus despite such mutations may due to the involvement of the host proteostasis network [1, 4].

The proteostasis network includes all the factors that control the function of proteins in their native state and minimize their non-functional or harmful reactions [5]. Many small proteins can achieve their folding even after the denaturators have been removed, due to the specific information sequence in their molecule; but those with more complex structures or multiple domains or subunits require regulators and/or mediators such as chaperones to achieve their main folding, otherwise they enter intracellular digestion processes such as proteasome or autophagy systems [6]. Thus, in a virus attack, on the one hand, the body’s immune system may be stimulated against the invading agent and chaperone molecules may have positive effects in this stimulation, and on the other side, the correcting and chaperoning molecular system may help the invader to survive. Viruses themselves do not have molecular chaperones and must use infected host chaperones [7]. Overexpression of host chaperones can be beneficial to the virus life cycle, including virus entry into the host cell, disassembly, activation of viral polymerases, transmission of the virus genome to the nucleus, as well as synthesis of structural proteins, the assembly, and the release of viral particles from the host cell. During these processes, both the cytoplasmic factor and the endoplasmic reticulum come into play. However, the capacity of the cell chaperoning activity is limited and mass production of viral proteins in infected host cells induces ER stress due to the production and accumulation of unfolded and misfolded viral proteins. ER stress induces the unfolded protein response (UPR), a process that aims to restore the ER homeostasis by suppressing translation and increasing the ER folding capacity, otherwise, disrupted cell proteosis leads to apoptosis or cell necrosis [7, 8].

It has been proved that bats are a repository for a variety of viruses, including the Ebola viruses, coronaviruses (CoVs), the Nipah and Hendra viruses [9]. Bats infected with these viruses are clinically asymptomatic, but these viruses often cause severe infections and mortality in mammals. For example, Ebola, a filovirus causes hemorrhagic disease, and CoVs cause severe acute respiratory syndrome (SARS), Middle East respiratory syndrome (MERS), porcine epidemic diarrhea (PED) and severe acute diarrhea syndrome (SADS). These observations have allowed researchers to speculate that bats are the likely reservoirs or ancestral hosts for several viruses. The reason for bats’ coexistence with viruses without being severely damaged may be due to new traits that bats have acquired during evolution. One of the reasons for bats’ resistance is probably due to the high expression of heat shock proteins in their various tissues, probably due to the metabolic stresses induced by high temperatures generated by their sustained flight, which causes cells to survive under stress [9, 10]. For example, the elevated HSP70 binds to and degrades the p65 subunit of NFκB in such conditions, and inhibits the “cytokine storm” [11] caused by viral infection such as SARS.

## The Proteostasis Network

During synthesis, the protein undergoes a variety of conformations until it reaches its most thermodynamically stable form by creating intramolecular interactions. Nevertheless, abnormal intramolecular interactions may occur that are thermodynamically more stable than the native state, leading to the formation of various species including oligomers, amorphous aggregates, and amyloid fibers [12]. The proteostasis network blocks such spontaneous interactions during and after translation, which may lead to problems such as Parkinson’s, Alzheimer’s, cardiomyopathy, and amyotrophic lateral sclerosis [13].

A proteostasis network consisting of ~ 2000 proteins in human cells is essential to control the normal state and functional levels of proteins to minimize potentially useless reactions or harmful pathways due to deformed or accumulated proteins [5]. The three arms of this network activity are protein synthesis, folding conformational maintenance and degradation. Different molecular chaperones cooperate with this network in folding, refolding and disaggregation reactions [5]. Chaperones can be considered as a functionally related group of proteins that help other macromolecules to achieve natural folding or proper assembly, under physiological and stress conditions. If the repair is useless, they lead the abnormal proteins to the cellular digestive system, the ubiquitin-proteasome system and the autophagosome-lysosomal machinery [14, 15]. In addition to correcting the physical structure of proteins, they mediate the solubility of misfolded proteins and prevent their self-association and toxic aggregation. However, the presence of various factors such as stress, mutation, heavy metals, reactive oxygen species, translational deviation or mRNA defect may overshadow cell repair operations and increase the possibility of abnormal intramolecular and/or intermolecular interactions [13, 16]. Chaperons typically recognize exposed hydrophobic amino acid residues and unstructured polypeptide backbones in their substrate proteins, which are unifying features of non-native conformations and make hydrophobic and electrostatic interactions with them to prevent (or reverse) misfolding and aggregation [16]. In this way, chaperones are similar to enzymes because they do not enter the structure of their substrate and remain intact after playing their role, but unlike enzymes, they lack an active site and work on a wide range of substrates.

In humans, ~ 300 chaperones have been identified that are metabolically divided into two main groups, ATP-dependent and non-ATP-dependent. Each group is also divided into subgroups based on their structure and function [17]. The cellular expression of some chaperones, known as heat-shock cognates (HSCs), is constitutive in order to perform vital housekeeping functions, while the expression of others termed as heat-shock proteins (HSPs) is due to existence of a stimulus or stress such as high heat or other forms of stress [18]. Chaperons are indicated by acronyms and the accompanying number indicates their molecular weight such as small heat shock proteins (sHSPs), HSP60, HSP70 and HSP90 [19]. HSPs, as their name implies, are produced in response to high temperatures, although they are also produced by other stressful stimuli such as cold. They are divided into classes of HSP33, HSP60, HSP70/HSP110, HSP90, HSP100, and the small heat-shock proteins (sHSPs) [20]. Among them, HSP70 and HSP90 are better known and their levels increase during viral infection. They are able to interact with vital components of both the innate and adaptive immune systems [21]. Therefore, some chaperones may have antiviral effects by stimulating the immune response against the virus. Guihur et al. suggested that following acute respiratory syndrome caused by coronavirus, HSP70 increased to minimize alveolar lung cell damage, but with the persistence of fever, chaperones decreased [22]. Therefore, the use of fever-relievers may enhance chaperones and improve their function in reducing apoptosis of respiratory cells.

## SARS-COV-2, and ER Stress

The severe acute respiratory syndrome coronavirus-2 (SARS-CoV-2), the causative agent of COVID-19 belongs to order Nidovirales, family Coronaviridae, and subfamily Orthocoronavirinae. Orthocoronavirinae consists of four genera, including Alphacoronavirus (α-CoV), Betacoronavirus (β-CoV), Gammacoronavirus (γ-CoV) and Deltacoronavirus (δ-CoV). Clinically, the important coronaviruses belong to the α-CoVs and β-CoVs genera that infect mostly mammals [23, 24].

**Fig. 1:**
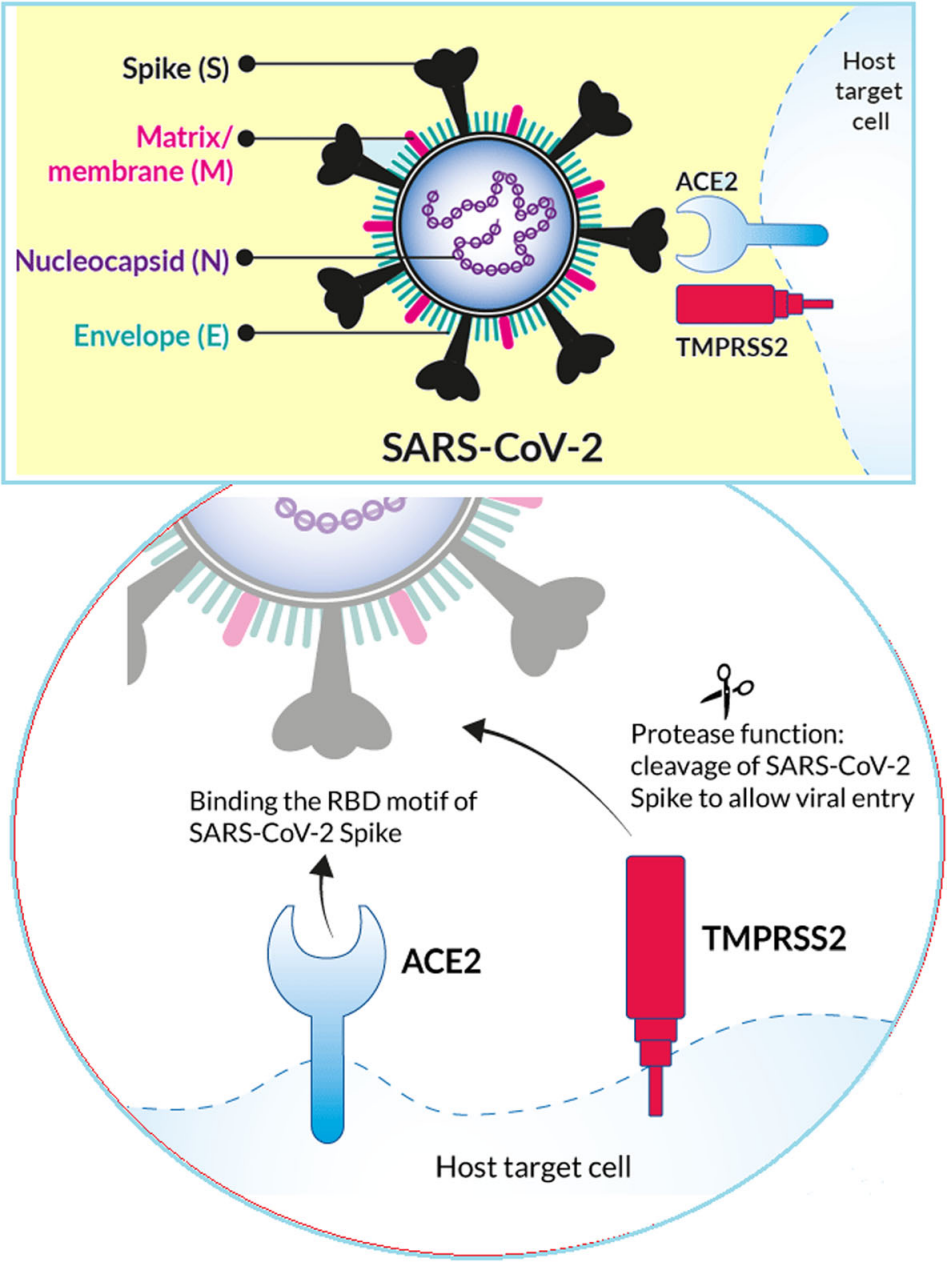
Spherical shape of coronavirus is characterized by heavily glycosylated spike (S), matrix or membrane (M) and envelope (E) proteins on the surface, and helical nucleocapsid inside which is formed by binding of the nucleocapsid (N) proteins on the genomic RNA. Receptors: ACE2 (angiotensin converting enzyme) and TMPRSS2 (Transmembrane protease, serine 2)

As it is shown in Fig. 1, SARS-CoV-2 from β-CoV genus, similar to other members of the Coronaviridae family, is an enveloped virus with the positive single-stranded RNA genome of around 26–32 Kb in size. It is methylated at 5′ and has a poly-A tail at 3′. The viral genome produces mRNAs that translate into four viral structural proteins, including the spike glycoprotein (S), membrane protein (M), nucleocapsid protein (N) and envelope protein (E). The virus uses the host protein synthesis apparatus to produce its proteins during the replication [25].

Glycoprotein S detects and binds to the host cell receptor on the membrane surface and is responsible for the virus entering the cell through endocytosis. It determines the invasive tissue and the host species due to its binding properties, which is determined by its receptor-binding domain (RBD). RBD or C-domain is responsible for identifying the host cell receptor and attaching the virus to it. Glycoprotein M is involved in the formation of envelope and assembly of the virus. The basic phosphoprotein N associates genomic RNA with the capsid [26]. During synthesis, newly synthesized viral polypeptides are checked by host chaperones such as calnexin and HSPs to ensure proper protein folding. For final maturity, the immature viral proteins in the ER are transported to the Golgi chamber, where the viral particles assemble to form complete viruses [27].

**Fig. 2:**
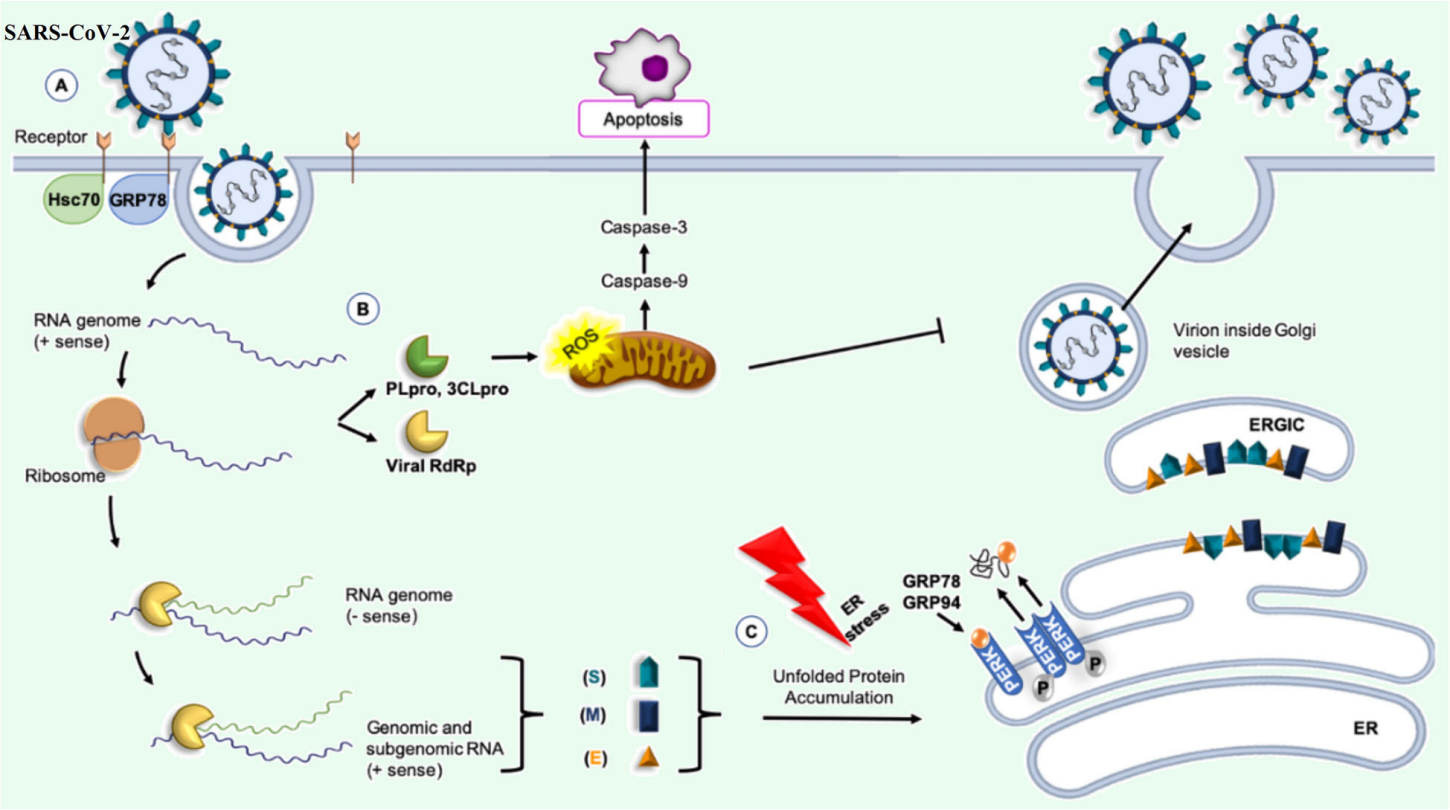
[7]: SARS-CoV-2 and molecular chaperones. **a** GRP78 or HSPA5 can be part of the ACE2-receptor complex recognized by the SARS-CoV-2 and can modulate virus entry. **b** ACE2-binding spike must be broken by specific proteases of 3CLpro (3C-like serine protease) or viral PLpro (papain-like cysteine protease) to allow the spike fusion domains to function. 3CLpro, also induces apoptosis thought caspase’s pathways. **c** Molecular chaperones, such as GRP78 and GRP94, participate in the folding of SARS-CoV-2 proteins. RdRp (RNA-dependentRNA polymerase)

As mentioned, glycoprotein S plays an important role in the entry of SARS-CoV-2 into host cells through ACE2 receptor located in the plasma membrane of host cells [25]. GRP78 (Glucose-regulated protein 78) or HSPA5 (Heat Shock Protein Family A (Hsp70) Member 5) is another receptor for spike glycoprotein on the cell surface and can mediate virus entry. This protein is also known as the immunoglobulin binding protein (BiP), which is an ER chaperone. Upon viral infection, GRP78 is upregulated, and translocated to the cell membrane where it is subjected to be recognized by the SARS-CoV-2 spike [28, 29] (Fig. 2).

Binding of the virus to cells occurs by binding of the viral spike protein to ACE2. The virus can then use endocytosis or fusion with the surface membrane to enter the host cells. In both cases, the ACE2-binding spike must be broken (“activated”) by specific protease of 3CLpro (3C-like serine protease) or viral PLpro (papain-like cysteine protease) to allow the spike fusion domains to function. 3CLpro, also induce apoptosis thought caspase’s pathway, causing a significant increase in reactive oxygen species (ROS) [30, 31]. In endosomes, the spike is broken down by Cathepsin L, then the viral membrane combines with the endocrine membrane [32]. At the cell surface, the spike is broken down by other proteases, mainly TMPRSS2 (transmembrane protease, serine 2), to ensure fusion of the virus membrane and host cell [33]. After fusion, the genomic and protein content of SARS-CoV-2 is transferred to the cytosol and the virus begins its replication cycle. The negative-sense genomic RNAs is synthesized by using the positive-sensegenome as a template and then translated by the host ribosome into the structural and accessory proteins [34].

Like other viruses, SARCE-Cov-2 is obligate intracellular parasite, and its replication requires host cell functions. CoVs rely absolutely on the protein synthesis machinery of the host cell [35]. Once a virus enters the cell, the RNA of the viral genome is translated to produce the viral proteins needed for subsequent RNA replication and transcription. Because genomic and subgenomic CoV mRNAs have a 5′ cap structure, most CoV mRNAs are thought to undergo cap-dependent translation using eIF4F. eIF4F and the 43S preinitiation complex access the 5′-end of the capped viral genome and the 43S preinitiation complex scans the 5′ UTR. The 80S complex is assembled at the translation initiation codon and protein synthesis starts and proceeds until the first termination codon is encountered and the ribosome dissociates from its mRNA template, resulting in polyprotein (pp) 1a [35–37]. Production of pp1ab (encoded by both ORF1a and ORF1b) requires a − 1 ribosomal frameshift before the translation stop codon is reached. This frameshift has been shown to occur in the overlap region between ORFs 1a and 1b. Viral proteins encoded downstream of ORFs 1a and 1b are not synthesized from the genome RNA but from a set of 5′-capped subgenomic mRNAs that carry the respective ORFs in their 5′-terminal regions. The two replicase polyproteins translated from ORFs 1a and 1b undergo proteolytic cleavage via viral-encoded proteinases encoded in ORF1a to generate 15–16 mature nonstructural proteins, termed nsp1 to nsp16. All of these nsps, except for nsp1. and nsp2 are considered essential for transcription and replication of CoV RNA [35, 36].

Rapid replication of the virus in the host cell is a sign of successful synthesis of its proteins in the rough endoplasmic reticulum, which is guaranteed by the use of ER molecular chaperones. Molecular chaperones, such as GRP78 and GRP94, BiP (binding immunoglobulin protein), PDI (protein disulfide isomerase), calreticulin, and calnexin participate in the folding of SARS-CoV-2 proteins, to ensure proper folding, sugar chain modification, and complex formation [38, 39]. BiP chaperones are attached to the ATF6 (activated transcription factor 6), IRE1 (inositol requiring kinase-1), and PERK (PKR-like ER related kinase) [29, 40]. The chaperone, also counteract the effect of the stress of the host cell caused by viral infection. However, high and uncontrolled expression of viral proteins increases the percentage of unfolded and misfolded proteins in ER lumen which results in induction of ER stress. ER stress dissociates BiP from ATF6, IRE1, and PERK leading to the activation of these membrane proteins and to an adaptive cellular response known as UPR. Free ATF6 is transported from the ER to the Golgi, where the amino portion of its cytoplasmic terminal is cleaved and converted into a factor transcription [41]. Then, it moves to the nucleus and induces transcription of genes involved in UPR, such as those encoding ER chaperones [42, 43]. After forming homo-multimer, IRE1 and PERK are autophosphorylated. IRE1 acts as an RNase and splices X-box binding protein-1 (XBP-1) mRNA which is translated into XBP-1. XBP-1 is a transcription factor that induces genes involved in the progression of the UPR and also mitogen-activated protein (MAP) kinases such as c-jun N-terminal kinase (JNK) [44]. PERK phosphorylates and inactivates eukaryotic initiation factor 2α (eIF2α), which suppresses protein synthesis and prevents from unfolded protein accumulation [45]. Therefore, UPR is a cell-protecting process managed by ER molecular chaperones, suppressing protein translation, and activating degradation of misfolded proteins. PERK also induces ATF4, which activates the transcription factor C/EBP homologous protein (CHOP). CHOP downregulates anti-apoptotic molecule of Bcl-2 which leads to apoptotic cell death. In ER stress, intracellular Ca^2+^ homeostasis is disrupted and Ca^2+^ released into the cytoplasm [46]. Calcium infiltration into the mitochondria makes release of cytochrome C, caspases 3 and 9 activation leading to apoptosis and cell death [47]. Therefore, in viral invasion and excessive load of unfolded or misfolded proteins, the UPR and ER-associated degradation (ERAD) prevents severe events in the body, and if necessary, end cell life. However, UPR may be useful for viral infection because ER chaperones increase and correct the folding of viral proteins, or may be harmful because increase in expression of agents involved in protein degradation may destroy viral proteins.

Patients infected with SARS-CoV2 have shown apoptosis in pneumocytes, T and B lymphocytes, and monocytes in the lungs, spleen, and lymph nodes. Thus, early in the course of the disease, they develop lymphopenia due to a decrease in the number of CD4+ and CD8+ T cells. ER stress plays an important role in the occurrence of these events [48, 49].

## ACE2 and ER Stress

ACE2 is found in a variety of cells and tissues, including the kidneys, lungs, heart, blood vessels, liver, and gastrointestinal tract and play an important role in preventing inflammation and tissue fibrosis. It is also abundant in the epithelium lining of the lungs, mouth and nose [50]. Previous studies have indicated that ACE2-mediated signaling reduces the effects of ER stress, so that, stimulation of ACE2 by agonists suppresses apoptosis mediated by ER stress [51]. Thus, occupying ACE2 by glycoprotein S may intensify ER stress via inhibiting ACE2 signaling. Increased ER stress may be one of the causes of pulmonary fibrosis in COVID 19 [52].

Comfortable and natural breathing in the lungs is due to the presence of surfactant that produced by type 2 pneumocytes and composed of phospholipids and pulmonary surfactant proteins of A, B, C, and D. ACE2 is highly expressed in type 2 pneumocytes, therefore, these cells are highly susceptible to infection and destruction by SARS-CoV-2, which is associated with decreased pulmonary surfactant and pneumonia [53, 54]. For this reason, surfactant replacement therapy was considered as one of the treatment suggestions to help improve the respiratory status of Covid 19 patients [55]. Moreover, surfactant protein C (SP-C) contains a chaperone-like of BRICHOS, which prevent SP-C from forming amyloid-like fibrils (Dolfe et al., 2016). In ER, BRICHOS of proSP-C binds to other SP-C peptides and prevent them from forming amyloid via inducing α-helix formation [56]. Decreased ACE2 signaling in COVID 19, and subsequently, increased ER stress and overt protein response, may disrupt such chaperone-like processes, causing fibrosis through SP-C accumulation in the lung.

The anti-inflammatory properties of ACE2 have been attributed to the inhibition of AngII (angiotensin II) production by increasing the conversion of AngI to other molecules, including Ang (1–9) to Ang (1–7). Ang (1–7), unlike AngII, is a vasodilator with anti-inflammatory properties, which acts through Mas receptors [25]. Therefore, ACE2 can be considered as a negative regulator of the local renin-angiotensin system in the lung, and its occupation in lungs by SARS-CoV-2 disrupts AngII tissue homeostasis, leading to inflammation and fibrosis. Moreover, increased AngII levels in the lungs constrict the alveoli, causing decreased O2 / CO2 exchange and decreased O2 levels in the blood. Decreased blood O2 level is life-threatening in patients with Covid 19 and necessitate the use of a ventilator apparatus [57].

Dipeptidyl peptidase 4 (DPP4) is another receptor for SARS-CoV-2, that its potency is enhanced by attaching to BiP. BiP is upregulated in ER stress. Thus, in SARS-CoV-2 infection, an increase in BiP molecules and its binding to DPP4 intensify the infection in patients with COVID-19 [58].

## SARS-COV-2, and Immune and Autoimmune Reactions

The most common fatal complication in COVID-19 patients is pneumonia and excessive systemic inflammation in which serum levels of pro-inflammatory cytokines such as IL-2, IL-6, IL-7, IL-10, MCP1, MIP1A, and TNFα increase [59]. High levels of inflammatory mediators in genetically predisposed individuals are due to over-stimulation of the immune system and over-activation of local macrophages by the corona virus. Activated macrophages, as the main immune cells in the lung parenchyma, may have an important role in the pathophysiology of SARS-CoV-2-induced acute respiratory distress syndrome (ARDS) [60]. Interestingly, although ACE2 is a membrane receptor for SARS-CoV-2 infection, it is also present in the bloodstream as a soluble protein and can bind to and inhibit SARS-CoV-2. Formation of SARS-CoV-2-ACE2 complex may stimulate immune system to produce antibodies [61].

Evidence from clinical trials has shown the presence of autoimmune reactions in COVID-19 patients and a strong association between this autoimmunity and severe SARS-CoV-2 infection [62]. Anti-nuclear autoantibodies (ANA) [63], Lupus anti-coagulant, Anti-SSA (anti–Sjögren’s-syndrome-related antigen A) autoantibodies, anti-cardiolipin (aCL) [64] and anti-β2 glycoprotein 1 (aβ2GP1) autoantibodies are autoimmune markers that have been detected in COVID-19 patients [65]. Cui et al. detected high blood factor VIII (FVIII), along with the last two antibodies, in COVID-19 patients, indicating hypercoagulation in them [66]. Bastard et al. reported the present of IgG auto-antibodies against type I IFNs in COVID-19 patients,, which destroy the ability of type I IFNs to prevent SARS-CoV-2 infection in vitro [67].

The melanoma differentiation-associated protein 5 (MDA5) is a receptor capable of detecting different type of RNA molecules [68]. Anti-MDA5 autoantibodies are associated with a rare disease of amyopathic dermatomyositis [69]. De Lorenzis et al. found anti-MDA5 antibody in SARS-CoV-2-infected patients [70].

Table 1 shows the types of autoantibodies found in COVID-19 patients, which increases the likelihood of additional antibodies. It also shows autoimmune disease/syndromes that are secondary to COVID-19 infection The production of such autoantibodies, in addition to being life-threatening, may be the cause of the variety of pathophysiological manifestations of the disease among individuals. The production of such antibodies, in addition to being life-threatening, may explain the variety of pathophysiological manifestations in COVID-19 patients.

**Table 1:** List of autoimmune diseases and autoantibodies associated with SARS-CoV-2 infection

Autoimmune disorder secondary to SARS-CoV-2 infection	Autoantibodies detected in COVID-19 patients
Antiphospholipid syndrome	Antiphosphatidylserine IgM/IgG
Immune thrombocytopaenic purpura	Anti-SSA autoantibody
Guillain-Barré syndrome	Anti-globulin antibodies
Cold agglutinin disease & autoimmune	Anti-nuclear antibodies (ANA)
Systemic lupus erythematosus (SLE)	Anti-cardiolipin (aCL) antibodies
Miler Fisher Syndrome (MFS)	Anti-MDA5 antibodies
Kawasaki disease	Anti-β2 glycoprotein 1 antibodies
NMDA-receptor encephalitis	LAC –lupus anticoagulant
Subacute thyroiditis	Antiprothrombin IgM
Graves’ disease	Anti-GD1b antibodies
Sarcoidosis	Anti-CCP antibodies
Myasthenia gravis	Anti-heparin PF4 complex antibody
Hemolytic anemia	Antiannexin V IgM/IgG
Neuromyelitis optica	pANCA AND cANCA
Large vessel vasculitis & thrombosis	
Type I diabetes	
Psoriasis	

Another case of autoimmunity phenomena may be due to molecular mimicry or immunological cross-reactivity in the immune response between viral and human molecules. The existence of such similarities may make it difficult for individuals to prepare and produce a vaccine and prescribe it, as autoimmune conditions may develop [71]. For example, in their bioinformatics study, Gammazza et al. showed the presence of the shared peptides between 17 human chaperones and viral proteins [72]. They stated that the cellular stress caused by SARS-CoV-2 could increase the expression of these chaperones and the possibility of their transfer from the intracellular site to the plasma membraneو that may stimulate the autoimmune reaction after detection by the immune system. For example, HSP60, HSP70, and HSP90 have been shown to be chaperones produced under stress and localized in plasma membranes [73].

## Chaperone Therapy

Although COVID-19 patients are treated with glucocorticoids to modulate the cytokine storm caused by severe immune stimulation by SARS-CoV-2, in some cases it is not sufficient. Due to the possible role of chaperones in the survival and infection of SARS-CoV-2, today chaperone therapy has attracted the attention of researchers to treat COVID-19 disease. Drug chaperones have been suggested to suppress cell dysfunction, inflammation, and apoptosis by preventing the accumulation of unfold proteins in ER and reducing ER stress, and have been proposed as a promising therapeutic strategy in COVID-19 [40]. However, this method of treatment is debatable. If the use of chaperone supplements helps to produce high-quality viral proteins as well as the stabilization of more compatible mutations, it causes the virus to multiply and survive in the patient; but if it stabilizes mutations that lead to disruption of the assembly of the virus and a reduction in the quality of its protein antennae, thereby reducing its infectivity, then it will be effective in treating COVID-19. For example, tauroursodeoxycholic acid (TUDCA) induces proper folding of functional proteins and improves proteostasis in virus-infected cells via a reduction in inflammation and cell apoptosis. The mentioned mechanism includes the suppression of BiP dissociation from PERK, and inhibition of eIF2α phosphorylation, and ATF4-CHOP activation. Reduced ATF4-CHOP increases the expression of anti-apoptotic BCL-2 and decreases the expression of pro-apoptotic Bax, and then suppression of caspase-3 [40]. The role of the drug chaperon of TUDCA against some acute diseases has been studied in cell cultures and animal models [74–76]. Another drug that can be considered in reducing the inflammatory effects of the coronavirus is the HSP60 inhibitor [77]. HSP60 is a vital chaperone for mitochondrial proteostasis that play a significant role in energy hemostasis through regulation of mitochondrial enzymes involved in oxidative phosphorylation. Under pathophysiological conditions the HSP60 can leave the mitochondria and excreted from the cell into circulation. In the blood stream, it can induce inflammatory response through binding to the Toll-like receptors, activating the NF-κB pathway and stimulating the proinflammatory cytokine release [77]. Jakovac et al. stated that after primary tissue damage by SARS-CoV-2, HSP60 could be released from damaged cells into the bloodstream [77]. Therefore, HSP60 inhibitors with potent immunosuppressive activity such as misoribine may be useful in inhibiting viral function, especially in patients suffering from hypertension and infected with SARS-CoV-2.

## Conclusions

Similar to the process of host protein synthesis, viruses typically exploit host chaperones to support viral transcription and replication. Such utilization of the host protection system helps the viruses to stabilize their mutations as well as the compatibility and persistence of the infection. Therefore, recognizing and then inhibiting the associated pathogenic chaperone and other factors in the proteostasis network that protects the viral life cycle and infection should be considered in the treatment of COVID19. Another contributing factor to SARS-CoV-2 infection is the similarity of the epitope to those in host. Such a phenomenon makes it difficult to produce a vaccine against SARS-CoV-2 and makes people suspicious of its use.

## Data Availability

Data presented in this manuscript is available upon request.

## Abbreviations

COVID-19: Coronavirus disease-19
SARS-CoV-2: Severe acute respiratory syndrome coronavirus 2
ACE2: Angiotensin-converting enzyme 2
HSPs: Heat shock proteins
RBD: Receptor-binding domain
PERK: PKR-like ER related kinase
HSV: Herpes simplex virus
CHOP: C/EBP homologous protein
